# Complex Geometry Strain Sensors Based on 3D Printed Nanocomposites: Spring, Three-Column Device and Footstep-Sensing Platform

**DOI:** 10.3390/nano11051106

**Published:** 2021-04-25

**Authors:** Alejandro Cortés, Xoan F. Sánchez-Romate, Alberto Jiménez-Suárez, Mónica Campo, Ali Esmaeili, Claudio Sbarufatti, Alejandro Ureña, Silvia G. Prolongo

**Affiliations:** 1 Materials Science and Engineering Area, Escuela Superior de Ciencias Experimentales y Tecnología, Universidad Rey Juan Carlos, C/Tulipán s/n, 28933 Móstoles, Madrid, Spain; xoan.fernandez.sanchezromate@urjc.es (X.F.S.-R.); monica.campo@urjc.es (M.C.); alejandro.urena@urjc.es (A.U.); silvia.gonzalez@urjc.es (S.G.P.); 2 Department of Mechanical Engineering, Politecnico di Milano, 20156 Milan, Italy; ali.esmaeili@polimi.it (A.E.); claudio.sbarufatti@polimi.it (C.S.)

**Keywords:** multifunctional composites, smart materials, sensing, 3D printing, carbon nanotubes

## Abstract

Electromechanical sensing devices, based on resins doped with carbon nanotubes, were developed by digital light processing (DLP) 3D printing technology in order to increase design freedom and identify new future and innovative applications. The analysis of electromechanical properties was carried out on specific sensors manufactured by DLP 3D printing technology with complex geometries: a spring, a three-column device and a footstep-sensing platform based on the three-column device. All of them show a great sensitivity of the measured electrical resistance to the applied load and high cyclic reproducibility, demonstrating their versatility and applicability to be implemented in numerous items in our daily lives or in industrial devices. Different types of carbon nanotubes—single-walled, double-walled and multi-walled CNTs (SWCNTs, DWCNTs, MWCNTs)—were used to evaluate the effect of their morphology on electrical and electromechanical performance. SWCNT- and DWCNT-doped nanocomposites presented a higher T_g_ compared with MWCNT-doped nanocomposites due to a lower UV light shielding effect. This phenomenon also justifies the decrease of nanocomposite T_g_ with the increase of CNT content in every case. The electromechanical analysis reveals that SWCNT- and DWCNT-doped nanocomposites show a higher electromechanical performance than nanocomposites doped with MWCNTs, with a slight increment of strain sensitivity in tensile conditions, but also a significant strain sensitivity gain at bending conditions.

## 1. Introduction

Structural health monitoring (SHM) refers to a set of technologies that allow one to detect, locate and quantify strain or damage appearance in a monitored structure [1]. There are several different techniques classified as active or passive methods depending on the requirement of an external stimulus. One of the most promising technologies is the use of self-sensing materials since the material itself is capable of acting as a sensor, which enables strain or damage detection throughout the whole material [2]. Self-sensing materials are commonly based on an insulating matrix doped with electrically conductive fillers. The changes on the load state of the material induce changes in the filler morphology and in the electrically conductive network as well. Thus, it is possible to detect these changes through a continuous measurement of the electrical resistance of the composite [3]. In this regard, carbon-nanoparticle-doped composites are widely studied for SHM purposes due to their excellent mechanical, electrical, thermal and piezoresistive properties [4]. In fact, there are several research studies about this topic with carbon nanofibers [5], carbon black [6], graphene nanoplatelets [7] and carbon nanotubes (CNTs) [8], among others.

More specifically, CNTs allow for increasing the electrical conductivity of the nanocomposite by several orders of magnitude by adding low filler contents (below 1 wt.%) [9]. There are different kinds of CNTs depending on their morphology, which are commonly classified as single-walled (SWCNTs), double-walled (DWCNTs) and multi-walled carbon nanotubes (MWCNTs) [10]. CNTs’ morphology could influence the electromechanical behavior of nanocomposites since they present different aspect ratios, which directly affect the electrically conductive networks created by CNTs, so the conductive mechanism may vary [11]. In this context, the tunneling conductive mechanism presents better strain-sensing properties than the intrinsic or contact-conductive mechanism between adjacent CNTs. Thus, it is important to obtain a well-dispersed material with low nanofiller content, i.e., near the percolation threshold region, the upper bound region, to reach maximum sensitivity. This is attributed to the predominant effect of tunneling resistance in governing piezoresistivity near the percolation threshold region [11]. Moreover, the addition of CNT to polymers also allows for other interesting functionalities, such as their Joule’s heating capability, which enables using them as anti-icing and de-icing systems [12,13], for self-healing [14,15,16] or for shape memory [17,18,19] purposes, among others.

On the other hand, 3D printing is receiving more and more attraction from the scientific community in the last few years due to the advantages it presents in comparison with traditional manufacturing techniques. The main advantages are the ability to manufacture unique pieces or limited batches at low cost, as well as allowing for the manufacture of pieces of great geometric complexity [20]. However, there are still important technological limitations with the 3D printing of nanocomposites with enhanced properties, such as electrical and thermal conductivity and self-heating, self-healing or self-sensing capabilities, among others. One of the main challenges is the UV light shielding effect caused by CNTs. CNTs are black, so they absorb part of the UV curing radiation. In fact, the increment of CNT percentage added and their dispersion degree on the mixture implies that less UV radiation is received by the photoinitiator, thus leading to a lower curing degree of the final nanocomposite [21,22,23].

In this paper, different complex geometry devices have been evaluated by applying different load requests to confirm their high self-sensing capabilities and therefore their wide applicability. The complex geometry sensors studied are a spring and a three-column device, while a footstep platform has been developed by using the three-column devices to confirm their applicability as useful in daily uses. These sensing devices are based on nanocomposites doped with three different types of CNTs (SWCNTs, DWCNTs and MWCNTs) and manufactured by digital light processing (DLP) 3D printing technology. In this regard, the present paper is a follow-up of previous work conducted with MWCNT-doped nanocomposites [24] to study how the addition of different CNT types (DWCNT and SWCNT) affect electrical and electromechanical properties and the strong relation of these properties to the dispersion state of the nanoparticles into the matrix.

## 2. Materials and Methods

### 2.1. Materials

The nanocomposite material used in this study is based on a commercial resin for stereolithography 3D printers doped with CNTs to provide electrically conductive pathways throughout the material.

The matrix, High Temp Resin supplied by Formlabs, is an acrylate-based resin designed for high temperature applications since it presents a heat deflection temperature using a 0.45 MPa load of 298 °C. Three different kinds of CNTs were added into the matrix in order to compare the effect of their morphology on the electrical and electromechanical properties of the printed nanocomposites: single wall carbon nanotubes with 3.96 wt.% OH content (SWCNTs), HDPlas single-wall/double wall carbon nanotubes (DWCNTs) from Cheaptubes and multi wall carbon nanotubes NC7000 (MWCNTs) from Nanocyl, whose main properties are shown in Table 1.

### 2.2. DLP Printing of Nanocomposites

**Figure 1:**
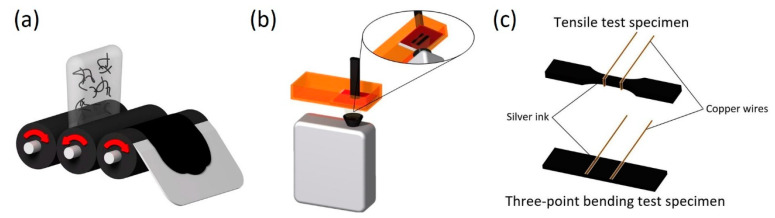
Manufacturing process of the nanocomposite specimens: (**a**) calendering dispersion process, (**b**) DLP 3D printing process and (**c**) electrode placement on 3D printed specimens.

A complete scheme of the manufacturing process of the nanocomposite specimens is shown in Figure 1. First, CNTs are added into the resin and manually mixed prior to performing a calendering dispersion process (Figure 1a) with an Exakt 80E machine by Exakt Technologies. In this three-roll milling dispersion technique, a seven-step procedure was applied, reducing the gap distance between rolls during each step, according to a previous study [25].

After completing the calendering stage, the mixture is poured into the resin vat of the 3D printer. This 3D printer, B9Creator by B9Creations, is based on DLP technology; the bottom surface of its resin vat is exposed to the light source from a digital projector with the desired 2D geometry, curing the first layer of the 3D model. Then, the build platform is raised to allow the uncured resin to fill the gap between the previously cured layer and the resin vat before curing the second layer. This process is repeated until the full part with the desired geometry is obtained (Figure 1b). The printing parameters used were 30 µm of layer thickness and 5.12 s of exposure time per layer. The exposure time was set to avoid sub- or overexposure, which could lead to geometrical distortions.

Specimens with different contents (0.050, 0.075 and 0.100 wt.%) of the three different CNT types (SWCNTs, DWCNTs and MWCNTs) were manufactured with different geometries, fulfilling the corresponding test standards: electrical conductivity (ASTM D257), tensile (ASTM D638) and three-point bending (ASTM D790) tests.

Once the test specimens are 3D printed, a UV post-curing treatment is performed for 30 min in a B9A-LCB-020 oven by B9Creations. Finally, copper wires are placed on the specimens as electrodes to perform the electromechanical tests, applying silver ink to reduce the electrical contact resistance between the electrodes and specimen. As shown in Figure 1c, in the case of tensile test specimens, the electrodes are placed all around the perimeter, while they are only placed on the tensile face for the three-point bending specimens.

### 2.3. Characterization

#### 2.3.1. Electrical Conductivity

DC volume conductivity tests were performed according to the ASTM D257 standard using a source-meter unit, Keithley 2410 From Keithley Instruments. The electrical conductivity was determined from the I-V slope, applying voltages from 0 to 50 V.

#### 2.3.2. Electromechanical Tests

The electromechanical behavior of the nanocomposite specimens was studied by performing tensile and three-point bending tests according to the ASTM D638 and D790 standards, respectively, in a Zwick Z100 universal test machine. In this regard, tensile tests were conducted at a 5 mm/min rate, while bending tests were carried out in two stages: the first at 1 mm/min up to 0.7 % strain in order to obtain the flexural modulus and the second at 10 mm/min up to specimen failure in order to determine the flexural strength. Electrical characterization was conducted simultaneously with the mechanical tests by monitoring the specimens’ electrical resistance changes between two copper wire electrodes (see Figure 1c) using an Agilent 33410A instrument. The strain sensitivity was calculated at low strain levels, up to 0.01 mm/mm, and was obtained by dividing the normalized electrical resistance by the applied strain, as shown in Equation (1):

(1) $$\mathrm{Strain}\;\mathrm{sensitivity}=\frac{\Delta R/R_{0}}{\varepsilon }$$

where R_0_ is the initial electrical resistance, ε is the strain in the sample and ΔR is the instantaneous electrical resistance increment with respect to the initial electrical resistance.

#### 2.3.3. Analysis of CNT Dispersion

Droplets of uncured CNT/resin dispersions were analyzed with a Leica optical microscope in transmitted light mode (TOM) equipped with a Nikon 990 camera in order to study the state of the nanoparticle dispersion. The area occupied by CNTs and the individual agglomerate size distribution were measured by digital image analysis to evaluate quantitatively the dispersion degree of each sample. These parameters allow us to understand how the dispersion state affects the curing process of the material and, furthermore, the electrical and electromechanical properties.

#### 2.3.4. Differential Scanning Calorimetry

Differential scanning calorimetry (DSC) tests were carried out with a Mettler Toledo 882e instrument, with non-isothermal DSC test conditions, from 0 to 300 °C at a rate of 10 °C/min in order to measure the glass transition temperature (T_g_) of the final cured specimens.

## 3. Results and Discussion

### 3.1. Electrical Conductivity

**Figure 2:**
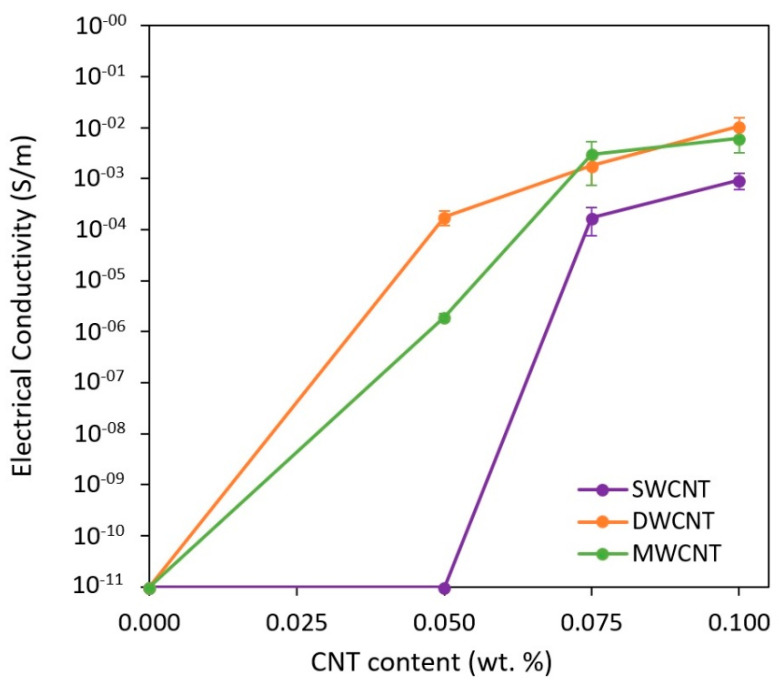
Electrical conductivity as a function of the type and content of CNTs.

Electrical conductivity values of nanocomposites doped with different CNT types— SWCNTs, DWCNTs and MWCNTs—are summarized in Figure 2. Here, several interesting factors can be observed. The electrical percolation threshold is different as a function of CNT geometry: below 0.050, around 0.050 and 0.075 wt. for nanocomposites doped with DWCNT, MWCNT and SWCNT, respectively. In addition, the final electrical conductivity of the nanocomposites at a higher CNT content where the electrical network is saturated is lower for the nanocomposites doped with SWCNTs.

This is directly related to the dispersion state of the material, the study of which will be developed in the next sections. As a summary, the electrical percolation threshold strongly depends on the dispersion state and the aspect ratio, which is inversely proportional to the percolation threshold [11], the aspect ratio of the different CNTs being 12,000 for SWCNTs, 10,000 for DWCNTs and 150 for MWCNTs. Therefore, the highest percolation threshold of the nanocomposite doped with SWCNTs is explained by the lowest dispersion degree, with large CNT aggregates. This means that they tend to form clusters of very entangled nanotubes. This entanglement leads to a reduction of effective aspect ratio due to a higher CNT waviness, as observed in previous studies [26,27]. This negatively affects the percolation threshold and the electrical conductivity, as there are not effective conductive pathways inside the material.

This means that the dispersion quality significantly affects the final properties. In this case, the poorer dispersion of SWCNTs induces a higher percolation threshold and a lower electrical conductivity of nanocomposites.

### 3.2. Structural Health Monitoring

**Figure 3:**
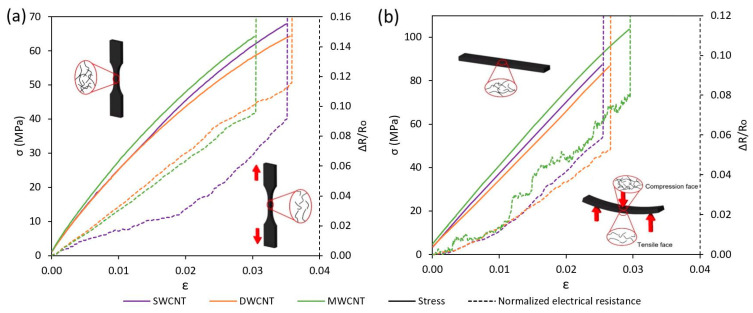
Examples of electromechanical tests for specimens doped with 0.1 wt.% CNTs in tensile (**a**) and three-point bending (**b**) load conditions.

First, Figure 3a,b show some examples of the electromechanical tests under tensile and three-point bending load conditions, respectively. Here, a gradual increase in the normalized electrical resistance with the applied strain on 3D printed specimens caused by the increase of the interparticle distance between adjacent CNTs can be observed. It is reflected in an increase of the electrical resistance associated with this effect, also known as tunneling resistance [28]. The sudden increase of the normalized resistance at the end of the test corresponds to the final failure of the sample.

**Figure 4:**
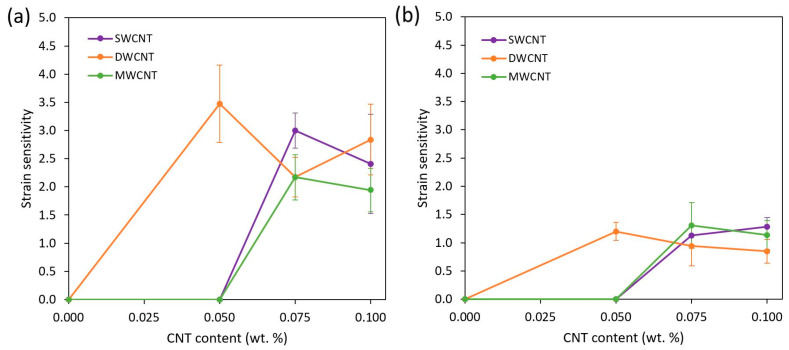
Strain sensitivity as a function of CNT content under tensile (**a**) and bending (**b**) load conditions.

On the other hand, Figure 4a,b summarize the values of the strain sensitivity for the tensile and three-point bending tests, respectively. It can be observed that the bending load gives significantly lower sensitivities with regard to tensile tests. This is explained by the combined effect of both tensile and compressive subjected faces in the case of the specimens subjected to a bending load. Here, the effect of compressive areas induces a decrease in the variation of electrical resistance due to applied strain, inducing a lower sensitivity when compared with tensile tests, where all the specimens are subjected to tensile strain [27,28]. Furthermore, a decreasing trend in the strain sensitivity can be observed when increasing the CNT content in every case. This is justified because near the percolation threshold, the tunneling effect is the most prevalent mechanism, as there is a high interparticle distance between nanofillers [27,28]. For this reason, every sample exhibits the highest sensitive response at these contents (here, it can be observed that MWCNT samples at 0.050 wt.% do not have high enough electrical conductivity to be monitored with the Agilent 33410A equipment). However, when increasing the content of the nanofiller, there are other mechanisms, such as contact between adjacent nanoparticles or even the intrinsic resistance, that can play a very dominant role in the electrical properties of the nanocomposite. The electrical resistance associated with the last phenomenon remains constant with applied strain, explaining the lower electrical sensitivities [29]. In this regard, the dispersion state plays a crucial role. Therefore, a better dispersed nanocomposite around the percolation threshold shows a prevalence of tunneling electrically conductive mechanisms among the CNTs leading to a higher sensitivity, whereas the electrical properties of a poorly dispersed nanocomposite are dominated by contact and intrinsic electrical resistances between CNTs inside the clusters, resulting in less sensitivity.

### 3.3. Sensitivity of Different Complex Geometry Sensors

**Figure 5:**
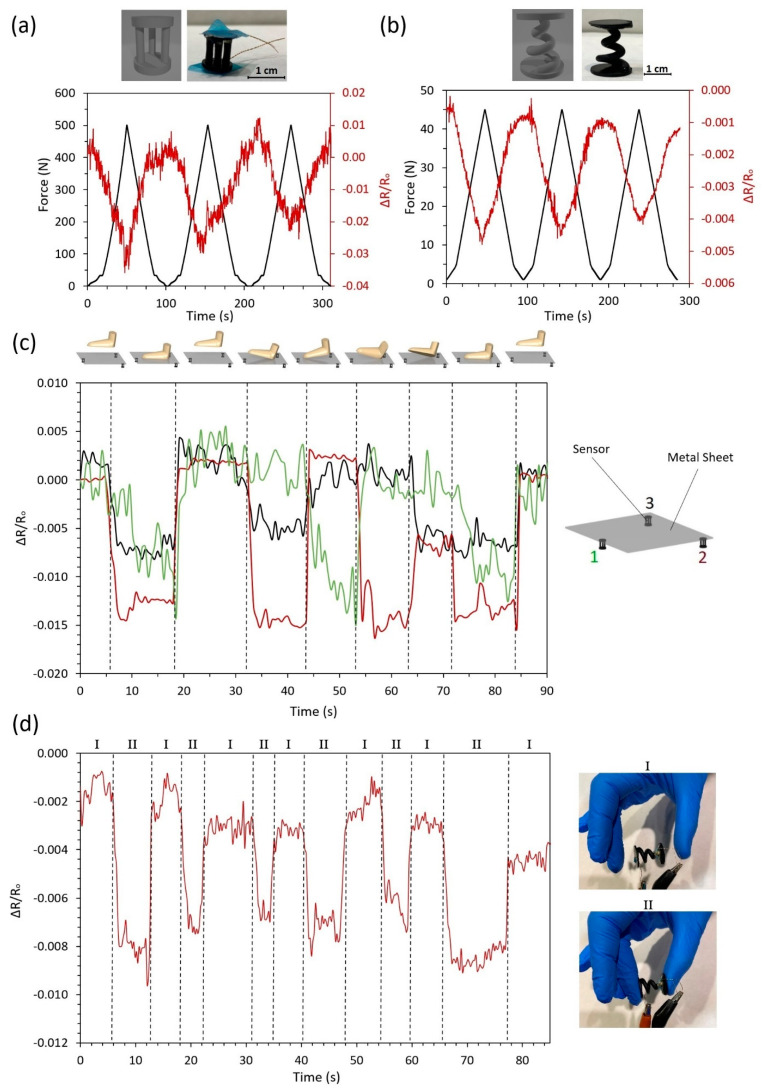
Self-sensing printed complex devices: (**a**) cycling compression load of the three-column device, (**b**) cycling compression load of the spring, (**c**) footstep platform and (**d**) manual compression of spring device.

An analysis of the electromechanical properties in complex elements manufactured by DLP technology is shown in the graphs of Figure 5. The main objective is to evaluate the applicability of these printed sensing materials for any device, tool, structure or item. Here, the most favorable condition regarding the electrical conductivity and strain sensitivity has been selected. In this regard, all the elements are made from resin doped with 0.100 wt.% DWCNTs. In addition, different types of loads are applied to analyze the performance and reproducibility of these sensing materials.

The electrical response under cycling loading of a three-column device (Figure 5a) and a spring (Figure 5b) is explored. In these examples, the 3D printed devices were subjected to compression loads, so a decrease of the electrical resistance is observed due to the reduction of the tunneling distance between adjacent nanoparticles. In addition, it can be observed that the electromechanical behavior is repeatable, reaching similar peak and valley values of the normalized electrical resistance in each cycle. The slight variations can be explained by the possible buckling of the structure at high load levels.

Furthermore, the sensitivity to load detection was investigated by a proof-of-concept under manual deformation. On the one hand, a footstep-sensing platform (Figure 5c) was developed to confirm the high applicability of these sensors in many daily uses. In this context, three three-column devices were placed below a metal platform and the sensitivity under different footstep configurations was analyzed. A total agreement between the variation of the electrical resistance of each sensor with the footstep performance was observed. For example, sensors 2 and 3 showed a drastic decrease of the electrical resistance when subjected to heel pressure, whereas sensor 1 was not affected. The different sensitivities among sensors depended on the applied force by the feet. In this regard, when comparing the initial and final situations, it can be concluded that the sensors were not damaged as they reached the same initial values.

Similarly, the spring configuration was tested with manual compression in order to prove its sensitivity (Figure 5d). It was observed that a drastic decrease of the electrical resistance was recorded when subjected to manual compression.

Therefore, the potential and applicability of the proposed materials as a pressure sensor have been proved. In this regard, the DLP technique could be a promising solution for the easy manufacturing of more complex devices, such as those shown in this section.

### 3.4. Morphology and Properties of Nanocomposites

All results previously shown confirm that the dispersion degree of CNTs in the nanocomposites significantly affects their electrical behavior and strain sensitivity. For this reason, a deep study of CNT dispersion state was carried out to optimize an experimental procedure to quantify the nanocomposite dispersion and morphology by digital image analysis of optical microscopy in transmitted light mode (TOM).

**Figure 6:**
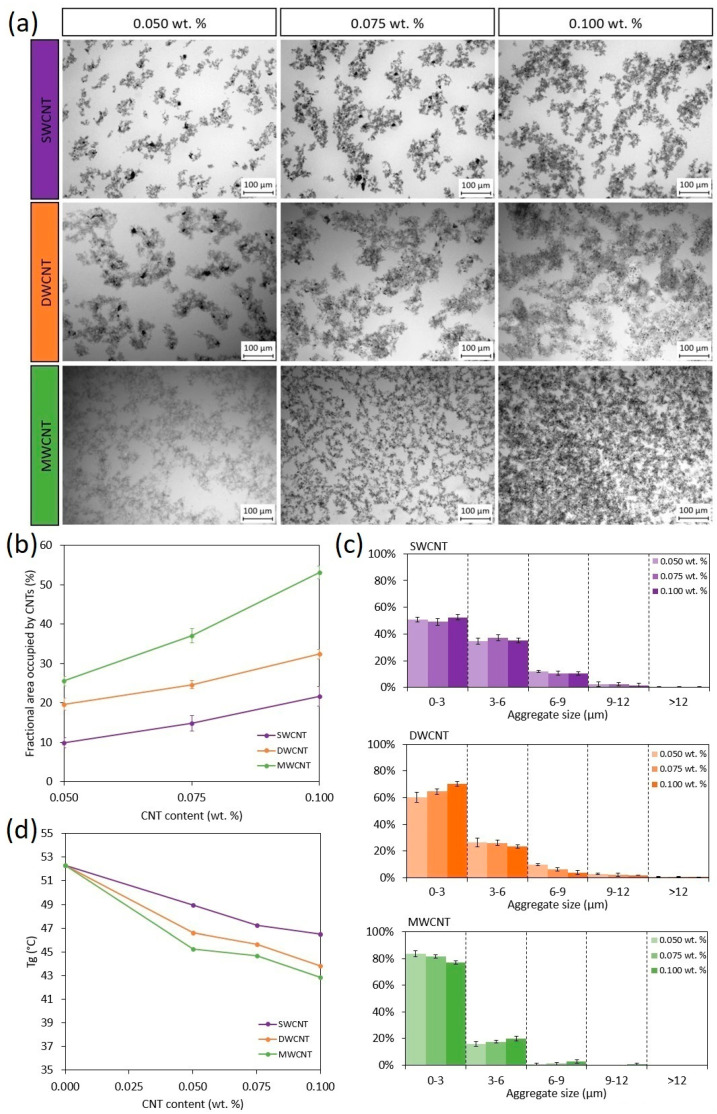
Dispersion degree and properties of printed nanocomposites: (**a**) TOM micrographs, (**b**) fractional area occupied by CNTs, (**c**) aggregate size distribution and (**d**) T_g_ of the nanocomposites.

Figure 6 shows the results obtained by image analysis of optical microscopies for the non-cured dispersions. First, in the micrographs of Figure 6a, it can be observed that the dispersion homogeneity is generally greater for the MWCNTs, followed by DWCNTs and SWCNTs, for each CNT content. This statement can be confirmed by image analysis, the results of which are summarized in Figure 6b, where the percentage of area occupied by nanotubes is shown, and Figure 6c, which collects the histograms of CNT aggregate size. In Figure 6b, it can be clearly observed that the area occupied by CNTs is much higher in the case of MWCNTs, followed by DWCNTs and SWCNTs. This behavior is explained by the fact that the CNT aggregate size is the lowest in the case of MWCNTs and the highest for SWCNTs, following the opposite trend. As expected, the formation of larger CNT aggregates induces a lower dispersion quality, reducing the percentage of area occupied, as occurs with SWCNTs. In contrast, the high dispersion degree obtained for the mixtures containing MWCNTs is confirmed by measuring a lower number of aggregates with a large size, thus showing a higher area occupied by CNTs. In this regard, the quality of the different studied dispersions is explained, considering the geometry of the individual CNTs. Other authors [26,30] have previously confirmed that SWCNTs and DWCNTs possess higher specific surface area (SSA) and larger aspect ratios compared to MWCNTs, inducing a higher tendency to aggregate. In this regard, the samples doped with CNTs with higher SSA and larger aspect ratios (around 10,000 for SWCNTs and DWCNTs) present poorer dispersions with larger CNT aggregates compared with the MWCNT doped ones, which present an aspect ratio of around 150.

The poor dispersions obtained for the samples doped with SWCNTs and DWCNTs are enhanced when increasing CNT content by decreasing the aggregate size. This can be explained by the effectiveness of three-roll milling method. It is widely known that, for the calendering process, a higher viscosity of the mixture allows for obtaining a better dispersion since the shear forces applied to the CNT aggregates are more effective [31,32]. On the other hand, in the case of MWCNT mixtures, which are already well dispersed even for the lowest content, an increase in the CNT content slightly increases the aggregate size [24].

Moreover, the dispersion state plays a crucial role in the final properties of the nanocomposites, especially for photocurable formulations. In this context, the area occupied by CNTs has a prevalent effect on the curing degree of the material. As mentioned before, CNTs are black-colored, so they absorb part of the UV radiation from the light source. This hinders photoinitiator UV absorption, leading to a lower curing degree. For this reason, the T_g_ of thermosetting resins decreases when increasing CNT content (Figure 6d), which is directly related to the increase in the area occupied by CNTs (Figure 6b). In addition, when comparing the different CNT types, the mixtures with the lowest degree of dispersion (SWCNT) also present the highest T_g_ values followed by the DWCNT and MWCNT ones, respectively, due to the same reason previously mentioned.

**Figure 7:**
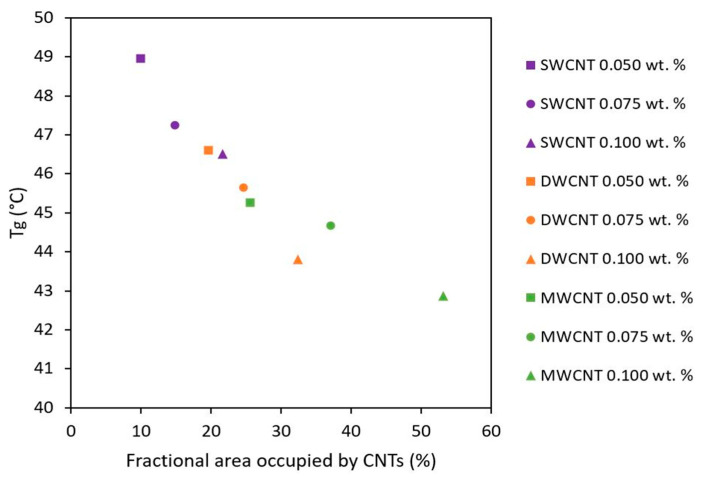
T_g_ as a function of fractional area occupied by CNTs.

Figure 7 confirms that there is a direct correlation between the T_g_ values of nanocomposites and the fractional area occupied by CNTs. The samples with a higher dispersion degree, measured by a higher percentage of area occupied by CNTs, present lower T_g_ values due to the hindering of the photocuring reaction. Therefore, this confirms that the CNTs’ geometry has an important influence on their dispersion, and this significantly affects the crosslinking degree for photocured resins. In this context, it is confirmed that the analysis of the CNT dispersion is essential in these systems to explain the final properties of nanocomposites. In this work, an easy experimental procedure, based on an image analysis of TOM micrographs, has been optimized to determine quantitatively the quality of CNT dispersions.

## 4. Conclusions

The self-sensing capabilities of nanocomposites obtained by digital light processing 3D printing technology using different types of CNTs were deeply studied. The strain-sensing tests revealed that the strain sensitivity is slightly higher for SWCNT and DWCNT doped nanocomposites than MWCNT doped ones, which depends on the dispersion state of the nanoparticles into the matrix and the number of effective electrical pathways that are created. In this regard, the strain sensitivity decreases when increasing the CNT content. Near the percolation threshold, the prevalence of a tunneling conductive mechanism over contact and intrinsic conductive mechanisms induces a higher strain sensitivity. Furthermore, the lower strain sensitivity showed by the specimens subjected to three-point bending load conditions compared with those tested in tensile conditions is explained by the effect of compressive areas, which induces a decrease of the variation of electrical resistance due to applied strain. Therefore, the role of nanofiller geometry and dispersion state on the electromechanical properties of DLP 3D printed nanocomposites has been determined.

The analysis of the electromechanical properties in more complex geometry parts also shows a great correspondence of electrical resistance to applied load, which demonstrates the versatility of DLP 3D printing technology for self-sensing devices.

It has also been confirmed that the analysis of CNT dispersion quality is essential to explain the behavior of printed nanocomposites since the dispersion degree, and thus the area occupied by CNTs, strongly affects the crosslinking degree of photopolymerized resin due to the UV absorption by graphitic nanofillers. In particular, the dispersion state of DWCNTs and SWCNTs is much more heterogeneous than in the case of MWCNTs, due to the higher aspect ratio of DWCNTs and SWCNTs compared with MWCNTs. In this regard, the better the dispersion, the higher the area occupied by CNTs, and thus, the lower the T_g_.

## Figures and Tables

**Table 1:** Properties of CNTs.

CNT Type	Supplier	Length (µm)	Diameter (nm)	Purity (wt.%)
SWCNTs	Cheaptubes	10–30	1–2	>98.5
DWCNTs	Cheaptubes	3–30	1–2	>99
MWCNTs	Nanocyl	1.5	9.5	90

## Data Availability

The data presented in this study are available on request from the corresponding author. The data are not publicly available due to technical limitations.

## References

[1] Giurgiutiu V. (2015). Structural health monitoring (SHM) of aerospace composites. Polymer Composites in the Aerospace Industry.

[2] Chung D.D.L. (2012). Carbon materials for structural self-sensing, electromagnetic shielding and thermal interfacing. Carbon N. Y..

[3] Han S., Meng Q., Xing K., Araby S., Yu Y., Mouritz A., Ma J. (2020). Epoxy/graphene film for lifecycle self-sensing and multifunctional applications. Compos. Sci. Technol..

[4] Llobet E. (2020). Carbon Nanomaterials.

[5] Wang Y., Wang Y., Wan B., Han B., Cai G., Li Z. (2018). Properties and mechanisms of self-sensing carbon nanofibers/epoxy composites for structural health monitoring. Compos. Struct..

[6] Dong W., Li W., Wang K., Guo Y., Sheng D., Shah S.P. (2020). Piezoresistivity enhancement of functional carbon black filled cement-based sensor using polypropylene fibre. Powder Technol..

[7] Sánchez M., Moriche R., Sánchez-Romate X.F., Prolongo S.G., Rams J., Ureña A. (2019). Effect of graphene nanoplatelets thickness on strain sensitivity of nanocomposites: A deeper theoretical to experimental analysis. Compos. Sci. Technol..

[8] Sánchez-Romate X.F., Moriche R., Jiménez-Suárez A., Sánchez M., Prolongo S.G., Güemes A., Ureña A. (2017). Highly sensitive strain gauges with carbon nanotubes: From bulk nanocomposites to multifunctional coatings for damage sensing. Appl. Surf. Sci..

[9] Mostaani F., Moghbeli M.R., Karimian H. (2018). Electrical conductivity, aging behavior, and electromagnetic interference (EMI) shielding properties of polyaniline/MWCNT nanocomposites. J. Thermoplast. Compos. Mater..

[10] Soni S.K., Thomas B., Kar V.R. (2020). A Comprehensive Review on CNTs and CNT-Reinforced Composites: Syntheses, Characteristics and Applications. Mater. Today Commun..

[11] Li B.J., Ma P.C., Chow W.S., To C.K., Tang B.Z., Kim J. (2007). Correlations between Percolation Threshold, Dispersion State, and Aspect Ratio of Carbon Nanotubes. Adv. Funct. Mater..

[12] Yao X., Hawkins S.C., Falzon B.G. (2018). An advanced anti-icing/de-icing system utilizing highly aligned carbon nanotube webs. Carbon N. Y..

[13] Hong J., Jung J.H., Yong S., Kim Y., Park J., Lee S.J., Choi J. (2020). Radio-frequency transparent carbon nanotube electrothermal film for radome de-icing application. J. Mater. Res. Technol..

[14] Ghosh T., Karak N. (2019). Multi-walled carbon nanotubes reinforced interpenetrating polymer network with ultrafast self-healing and anti-icing attributes. J. Colloid Interface Sci..

[15] Jiménez-Suárez A., Martín-González J., Sánchez-Romate X.F., Prolongo S.G. (2020). Carbon nanotubes to enable autonomous and volumetric self-heating in epoxy/polycaprolactone blends. Compos. Sci. Technol..

[16] Wang H., Yang Y., Zhang M., Wang Q., Xia K., Yin Z., Wei Y., Ji Y., Zhang Y. (2020). Electricity-Triggered Self-Healing of Conductive and Thermostable Vitrimer Enabled by Paving Aligned Carbon Nanotubes. ACS Appl. Mater. Interfaces.

[17] Li M.Q., Wu J.M., Song F., Li D.D., Wang X.L., Chen L., Wang Y.Z. (2019). Flexible and electro-induced shape memory Poly(Lactic Acid)-based material constructed by inserting a main-chain liquid crystalline and selective localization of carbon nanotubes. Compos. Sci. Technol..

[18] Raja M., Ryu S.H., Shanmugharaj A.M. (2014). Influence of surface modified multiwalled carbon nanotubes on the mechanical and electroactive shape memory properties of polyurethane (PU)/poly(vinylidene diflouride) (PVDF) composites. Colloids Surfaces A Physicochem. Eng. Asp..

[19] Li Z., Qi X., Xu L., Lu H., Wang W., Jin X., MD Z.I., Zhu Y., Fu Y., Ni Q.-Q. (2020). A Self-Repairing, Large Linear Working Range Shape Memory Carbon Nanotubes/Ethylene Vinyl Acetate Fiber Strain Sensor for Human Movement Monitoring. ACS Appl. Mater. Interfaces.

[20] Singh R., Singh S., Nanak G., Engineering D. (2017). Additive Manufacturing: An Overview.

[21] Gonzalez G., Chiappone A., Roppolo I., Fantino E., Bertana V., Perrucci F., Scaltrito L., Pirri F., Sangermano M. (2017). Development of 3D printable formulations containing CNT with enhanced electrical properties. Polymer.

[22] Mu Q., Wang L., Dunn C.K., Kuang X., Duan F., Zhang Z., Qi J.H., Wang T. (2017). Digital light processing 3D printing of conductive complex structures Digital light processing 3D printing of conductive complex structures. Addit. Manuf..

[23] Sandoval J.H., Wicker R.B. (2006). Functionalizing stereolithography resins: Effects of dispersed multi-walled carbon nanotubes on physical properties. Rapid Prototyp. J..

[24] Cortés A., Sánchez-Romate X.F., Jiménez-Suárez A., Campo M., Ureña A., Prolongo S.G. (2020). Mechanical and strain-sensing capabilities of carbon nanotube reinforced composites by digital light processing 3D printing technology. Polymers.

[25] Jiménez-Suárez A., Campo M., Sánchez M., Romón C., Ureña A. (2012). Dispersion of carbon nanofibres in a low viscosity resin by calendering process to manufacture multiscale composites by VARIM. Compos. Part B Eng..

[26] Sánchez-Romate X.F., Jiménez-Suárez A., Sánchez M., Güemes A., Ureña A. (2016). Novel approach to percolation threshold on electrical conductivity of carbon nanotube reinforced nanocomposites. RSC Adv..

[27] Esmaeili A., Sbarufatti C., Ma D., Manes A., Jiménez-Suárez A., Ureña A., Dellasega D., Hamouda A.M.S. (2020). Strain and crack growth sensing capability of SWCNT reinforced epoxy in tensile and mode I fracture tests. Compos. Sci. Technol..

[28] Esmaeili A., Sbarufatti C., Jiménez-Suárez A., Urena A., Hamouda A.M. (2020). Piezoresistive characterization of epoxy based nanocomposites loaded with SWCNTs-DWCNTs in tensile and fracture tests. Polym. Compos..

[29] Sánchez-Romate X.F., Artigas J., Jiménez-suárez A., Sánchez M., Güemes A., Ureña A. (2019). Critical parameters of carbon nanotube reinforced composites for structural health monitoring applications: Empirical results versus theoretical predictions. Compos. Sci. Technol..

[30] Gojny F.H., Wichmann M.H.G., Fiedler B., Schulte K. (2005). Influence of different carbon nanotubes on the mechanical properties of epoxy matrix composites—A comparative study. Compos. Sci. Technol..

[31] Fan Z., Advani S.G. (2007). Rheology of multiwall carbon nanotube suspensions. J. Rheol..

[32] Battisti A., Skordos A.A., Partridge I.K. (2009). Monitoring dispersion of carbon nanotubes in a thermosetting polyester resin. Compos. Sci. Technol..

